# Spontaneous Resolution of Unexplained Neutropenia in Primary Hyperparathyroidism: A Case Report

**DOI:** 10.7759/cureus.98094

**Published:** 2025-11-29

**Authors:** Zin Aung Soe, Eleni E Ladikou, Joel A Newman

**Affiliations:** 1 Haematology, Eastbourne District General Hospital - East Sussex Healthcare NHS Trust, Eastbourne, GBR

**Keywords:** adult primary hyperparathyroidism, myelodysplastic syndrome (mds), pediatric primary hyperparathyroidism, refractory pancytopenia, reversible neutropenia, unexplained neutropenia

## Abstract

A 65-year-old gentleman was referred to our institution through the two-week-wait pathway after presenting with a four-week history of bilateral lymphadenopathy. A lymph node biopsy confirmed the diagnosis of follicular lymphoma, grade III, for which he was treated with six cycles of obinutuzumab and bendamustine. A completion computed tomography scan showed stable appearances of the spleen and stranding in the left iliac chain. Obinutuzumab was continued as maintenance therapy. Despite the completion of lymphoma treatment and without evidence of disease relapse, he developed a persistent neutropenia, leading to recurrent chest infections and oesophageal candidiasis. He underwent multiple bone marrow biopsies to investigate further. During the same timeline, he was concurrently diagnosed with primary hyperparathyroidism (PHPT). Parathyroidectomy was performed following the recommendation from the Endocrine Multidisciplinary Team review, after initial delays due to concerns about operating while neutropenic. Following parathyroidectomy, the hypocalcaemia-related symptoms resolved, and the serum calcium and parathyroid hormone (PTH) levels have returned to normal. Notably, a complete restoration of the neutropenia was observed, along with the recovery of chronic persistent respiratory infections.

This case highlights that isolated neutropenia can also be associated with PHPT, providing further evidence, in addition to existing research and similar case reports, that have focused more on anaemia and pancytopenia. Therefore, we recommend investigating refractory neutropenia or pancytopenia with bone profiles, including calcium, phosphate, and PTH levels. Early identification of hyperparathyroidism and prompt referral to the Endocrinology Team can be critical, as surgical intervention can lead to a complete recovery of neutropenia.

## Introduction

There have been previous reports of pancytopenia associated with primary hyperparathyroidism (PHPT), but isolated neutropenia has rarely been reported. In this report, we present a case of reversible neutropenia in a patient with PHPT, and discuss the clinical implications for managing patients with unexplained neutropenia. 

PHPT is an endocrine disorder characterized by the autonomous, excessive secretion of parathyroid hormone (PTH), leading to a dysregulation of calcium homeostasis [1]. The predominant aetiology, accounting for approximately 85% of cases, is a single benign parathyroid adenoma, which is most often sporadic [2]. Less frequently, the condition arises from multiple adenomas or four‑gland hyperplasia.

The clinical presentation of PHPT is categorized into the following distinct phenotypes [2]: (i) symptomatic PHPT - which manifests with overt complications, including renal (e.g., nephrolithiasis, chronic kidney disease) and skeletal (e.g., osteitis fibrosa cystica, fractures); (ii) asymptomatic PHPT - diagnosed incidentally through biochemical tests, with few or no overt symptoms. This category can be further subdivided based on the presence or absence of subclinical target organ involvement; (iii) normocalcemic PHPT - a variant wherein patients have elevated PTH levels with consistently normal serum calcium, with or without associated skeletal or renal complications.

PHPT is diagnosed biochemically by the simultaneous finding of hypercalcemia and either an elevated or inappropriately normal (non-suppressed) plasma PTH level. While imaging (e.g., sestamibi scan and ultrasound) is not required for the diagnosis, it is a valuable tool for surgical planning [3].

Parathyroidectomy is the definitive treatment for PHPT. It is indicated for all symptomatic patients, and is recommended for many asymptomatic patients who meet specific criteria. For asymptomatic patients who do not meet surgical indications, decline surgery, or are poor surgical candidates, surveillance and medical management are appropriate [1].

Neutrophils are white blood cells that play a critical role in the immune system, and impairment in their quantity or quality can predispose individuals to severe and potentially life‑threatening infections.

The clinical severity of neutrophil depletion can be classified by the absolute neutrophil count (ANC), a calculated value derived from the total white blood cell count and the percentage of polymorphonuclear cells and band forms [4]. The standard threshold for neutropenia is an ANC below 1.5 × 10^9^/L, which is further graded by severity: mild, ANC 1.0-1.5 × 10^9^/L; moderate, ANC 0.5-0.99 × 10^9^/L; severe, ANC 0.2-0.49 × 10^9^/L; very severe, ANC < 0.2 × 10^9^/L.

It is imperative to note that the ANC is not a fixed value for everyone, and demonstrates physiological variation based on factors such as age and certain ethnicities. The existence of a lower normal baseline in certain healthy individuals is a crucial consideration for accurate diagnosis, to avoid overdiagnosis of neutropenia. 

The pathophysiological mechanisms underlying neutropenia can be categorized into decreased bone marrow production, accelerated peripheral utilization or destruction, and altered margination. These mechanisms stem from either congenital or acquired aetiologies. In the adult population, infectious diseases and drug-induced myelosuppression are the most common causes. Other common acquired causes include bone marrow infiltration or failure (e.g., leukaemia and aplastic anaemia), and nutritional deficiencies (e.g., vitamin B12 and folate). Congenital syndromes are comparatively rare.

The diagnostic confirmation of neutropenia requires a repeat complete blood count (CBC) with differential, and manual peripheral blood smear review, to rule out analytical artefacts and identify characteristic morphological abnormalities. In cases of suspected drug-induced neutropenia, discontinuation of the causative agent is both diagnostic and therapeutic, typically leading to count recovery within one to three weeks, without the need for any further investigation. However, in the meantime, a diagnostic work-up should be done to exclude other causes, particularly underlying infections such as HIV, viral hepatitis, and other viral syndromes [4].

## Case presentation

Medical background and presentation 

A 65-year-old gentleman was referred to our institution through the two-week-wait pathway after presenting with a four-week history of bilateral lymphadenopathy. He has a past medical history of type 1 diabetes, Dupuytren’s contracture, vitreous haemorrhage, and Charcot arthropathy of the right leg. He has no known drug allergies, and his regular medications are atorvastatin, Toujeo insulin, and Humalog insulin. He reports a family history of Dupuytren’s contracture.

On examination at the time of presentation, there were palpable lymph nodes in the cervical, supraclavicular, and axillary regions, as well as bilaterally in the inguinal regions. Notably, an obvious lymph node mass was present in the right groin, extending to the upper thigh. His abdomen was soft and non-tender, but bulky. However, there was no clinically palpable hepatomegaly. 

**Figure 1:**
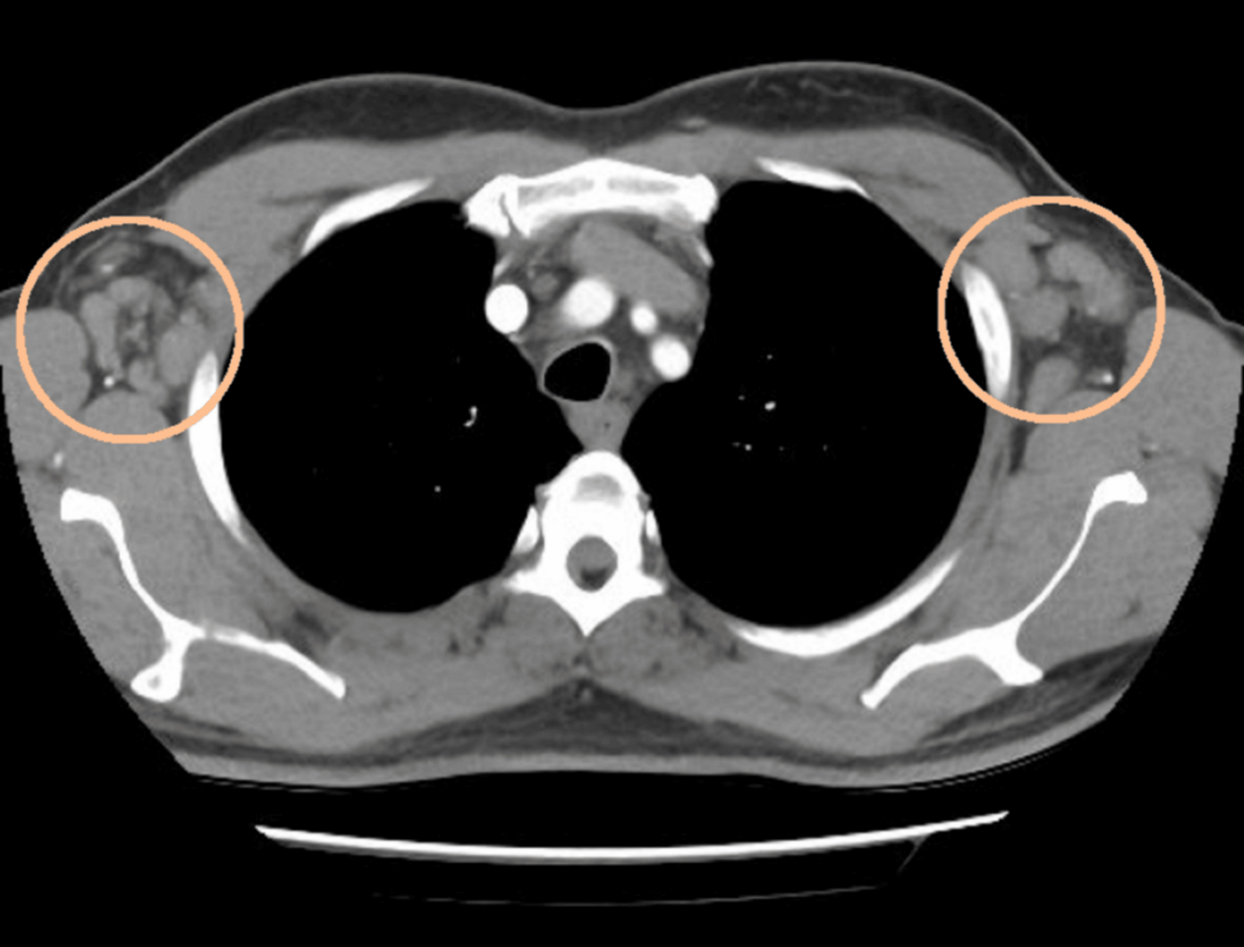
PET scan showing bilateral axillary lymphadenopathy This is the PET scan showing bilateral axillary lymphadenopathy - performed at the time of referral from the General Practitioner with presentation of the lymphadenopathy. PET: positron emission tomography

**Figure 2:**
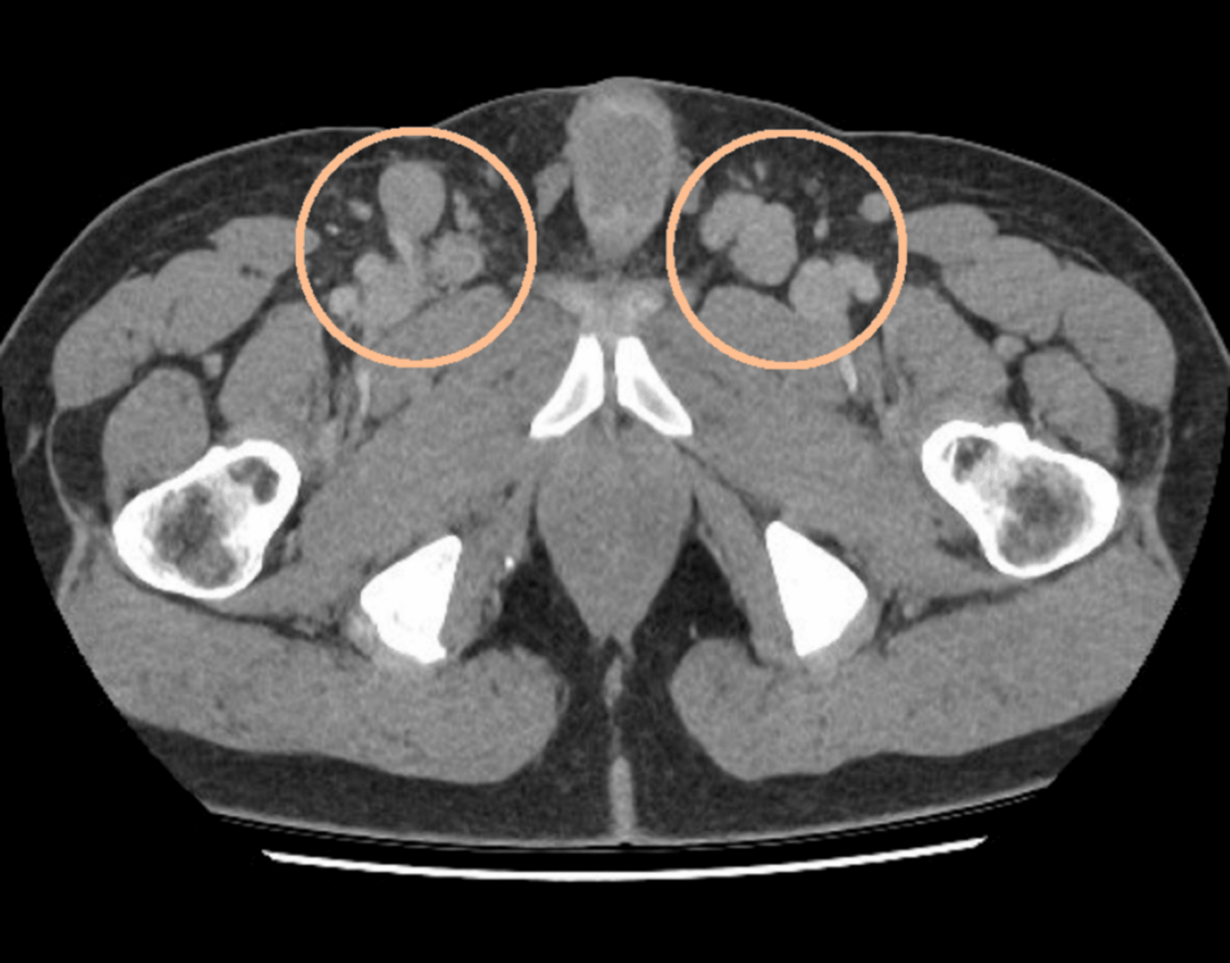
PET scan showing bilateral groin lymphadenopathy This is the PET scan showing bilateral groin lymphadenopathy - performed at the time of referral from the General Practitioner with presentation of the lymphadenopathy. PET: positron emission tomography

**Figure 3:**
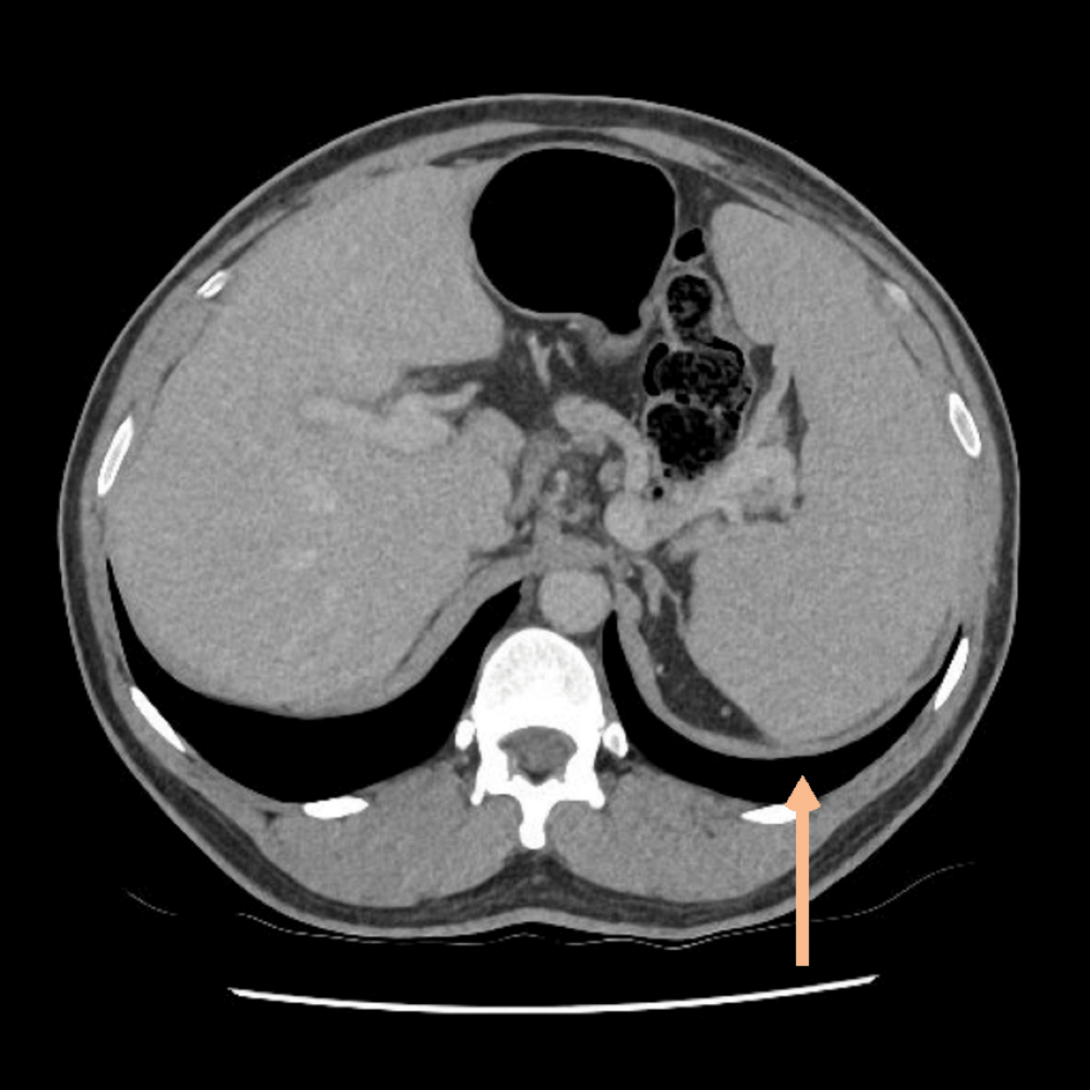
PET scan showing splenomegaly This is the PET scan showing splenomegaly - performed at the time of referral from the General Practitioner with presentation of the lymphadenopathy. PET: positron emission tomography

A positron emission tomography (PET) scan confirmed the presence of lymphadenopathy above and below the diaphragm (Figures 1-2), splenic involvement (Figure 3), and diffuse marrow changes likely to be reactive.

A lymph node biopsy confirmed the diagnosis of follicular lymphoma, grade III, for which he was treated with six cycles of obinutuzumab and bendamustine. A completion computed tomography (CT) scan showed stable appearances of the spleen and stranding in the left iliac chain. Obinutuzumab was continued as maintenance therapy. Following this, he developed a persistent, unexplained neutropenia, leading to recurrent chest infections and oesophageal candidiasis. 

Investigation and management for neutropenia 

As such, maintenance obinutuzumab was subsequently withheld, and the underlying cause of neutropenia was investigated. He underwent multiple bone marrow biopsies. 

The first two biopsies did not show any evidence of underlying lymphomatous disease, but showed granulocytic maturation arrest. This was initially thought to be an autoimmune phenomenon, given his history of type 1 diabetes, though drug-induced neutropenia is also considered. Acyclovir and co-trimoxazole, which were part of the prophylactic regimen, were also discontinued alongside obinutuzumab. In addition to the granulocyte‑colony stimulating factor (G‑CSF), a short course of steroids and two doses of high‑dose immunoglobulin replacement were trialled; however, there was no improvement in the neutrophil count. To exclude other common causes of neutropenia, further investigations were performed. Viral markers of HIV, hepatitis B surface antigen, and hepatitis C Ab returned negative. No aetiology that could explain isolated neutropenia was identified. 

The third bone marrow biopsy showed morphological changes consistent with a myelodysplastic syndrome with single-lineage dysplasia (MDS‑SLD). This was also noted on immunophenotyping. However, the myeloid gene panel was negative for abnormalities. 

During the timeline of follow-up and investigation for the neutropenia, the patient was concurrently diagnosed with PHPT. Following the review by the Parathyroid Multidisciplinary Team, a surgical referral was made. He then underwent a minimally invasive right parathyroidectomy, after initial delays due to concerns about operating while neutropenic. 

Outcome and follow-up

Histology confirmed that the resected parathyroid gland was a benign parathyroid adenoma. Following parathyroidectomy, the hypocalcaemia-related symptoms resolved, and the serum calcium and PTH levels normalized. Notably, a complete restoration of the neutropenia was observed, along with recovery from chronic persistent infections.

**Figure 4:**
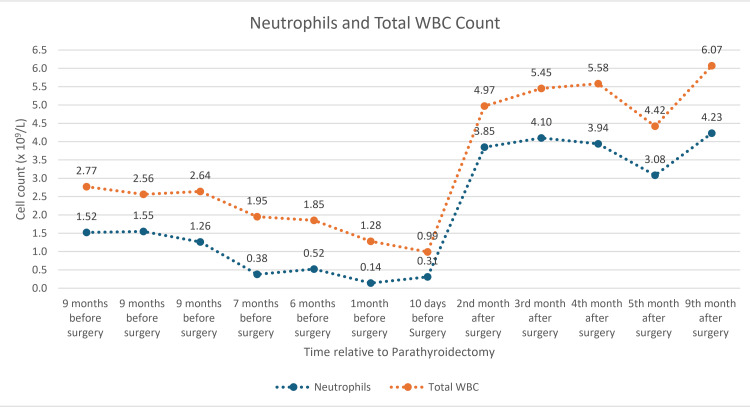
WBC and neutrophil chart This is the graph showing the patient's neutrophil and total WBC counts before and after parathyroid surgery. The graph clearly demonstrates the resolution of neutropenia and the recovery of WBC counts following parathyroid surgery for primary hyperparathyroidism.

At subsequent follow-up appointments, this improvement in neutropenia has been sustained for up to nine months after parathyroidectomy (Table 1 and Figure 4). This association explains hyperparathyroidism as the cause of the neutropenia.

**Table 1:** Neutropenia and leukopenia table These are the full blood count results before and after the parathyroid surgery. The results outside the standard reference range are marked - ↑ meaning higher than the normal range, and ↓ meaning lower than the normal range.

	Normal reference range	Nine months before surgery	Nine months before surgery	Nine months before surgery	Seven months before surgery	Six months before surgery	One month before surgery	Ten days before Surgery	Second month after surgery	Third month after surgery	Fourth month after surgery	Fifth month after surgery	Ninth month after surgery
Haemoglobin	130 - 180	102 ↓	93 ↓	99 ↓	121 ↓	118 ↓	133	131	118 ↓	120 ↓	126 ↓	134	140
White Cell Count	4.0 - 11.0	2.77 ↓	2.56 ↓	2.64 ↓	1.95 ↓	1.85 ↓	1.28 ↓	0.99 ↓	4.97	5.45	5.58	4.42	6.07
Platelet Count	150 - 400	382	425 ↑	453 ↑	289	355	218	220	278	305	301	265	234
Haematocrit	0.37 - 0.51	0.315 ↓	0.312 ↓	0.332 ↓	0.372	0.383	0.428	0.398	0.373	0.376	0.405	0.418	0.438
Mean Cell Volume	80 - 100	66.9 ↓	70.7 ↓	70.8 ↓	74.1 ↓	77.4 ↓	79.1 ↓	76.7 ↓	80.7	80.7	84.6	82.6	84.4
Red Cell Count	4.5 - 6.5	4.71	4.41 ↓	4.69	5.02	4.95	5.41	5.19	4.62	4.66	4.79	5.06	5.19
Mean Cell Haemoglobin	27 - 32	21.7 ↓	21.1 ↓	21.1 ↓	24.1 ↓	23.8 ↓	24.6 ↓	25.2 ↓	25.5 ↓	25.8 ↓	26.3 ↓	26.5 ↓	27
Red Cell Distribution Width	11.8 - 14.8	18.3 ↑	18.4 ↑	19.0 ↑	18.5 ↑	15.2 ↑	19.3 ↑	17.5 ↑	17.2 ↑	17.6 ↑	16.7 ↑	14.2	13.9
Neutrophil	2 - 7.5	1.52 ↓	1.55 ↓	1.26 ↓	0.38 ↓	0.52 ↓	0.14 ↓	0.31 ↓	3.85	4.1	3.94	3.08	4.23
Erythrocyte Sedimentation Rate	1 - 10	-	-	-	-	-	-	-	52 ↑	40 ↑	39 ↑	11 ↑	9

## Discussion

We report an unusual case of a 65-year-old gentleman with PHPT who presented with persistent neutropenia following the treatment of lymphoma. Despite the completion of lymphoma treatment, with no evidence of disease relapse, his neutropenia persisted, and he underwent a bone marrow biopsy to investigate further. The bone marrow biopsy showed morphological changes consistent with MDS‑SLD. A drug-related aetiology was considered, and suspected medications were discontinued. However, there was no improvement in the neutrophil count despite this, and no response was noted again with the trial of G‑CSF, a short course of steroids, and two doses of immunoglobulin. Viral screening tests came back negative. Concurrently, he was found to have PHPT. Parathyroidectomy was performed following the recommendation of the Endocrine Multidisciplinary Team, despite the neutropenic state. Histology confirmed that the resected parathyroid gland was a benign parathyroid adenoma. Post-operatively, the hypocalcaemia-related symptoms resolved, and the serum calcium and PTH levels normalized. This was accompanied by a complete restoration of neutrophil counts and the resolution of the chronic recurrent infections. 

The common presentations of PHPT are mainly skeletal and muscular manifestations due to hypercalcaemia. Neutropenia is a common haematological abnormality, with multiple possible causes; however, it is not typically associated with hyperparathyroidism. This can, however, rarely occur as part of pancytopenia, which has been infrequently reported in hyperparathyroidism. Based on previous case reports, there are only a few cases of hyperparathyroidism leading to bone marrow fibrosis, causing subsequent pancytopenia. Our case is unique in that it involved isolated neutropenia, without anaemia or thrombocytopenia, and the bone marrow biopsy showed the features of SLD, without evidence of bone marrow fibrosis.

MDS, also termed myelodysplastic neoplasms, constitute a group of clonal haematopoietic stem cell disorders characterized by ineffective hematopoiesis, causing cytopenias and a variable risk of progression to acute myeloid leukaemia (AML). The disease is most prevalent in older adults. Its presentation in children and younger adults is less common, and is frequently associated with underlying congenital syndromes, or is secondary to predisposing factors such as prior chemotherapy, radiation exposure, or genetic predisposition [5]. Bone marrow biopsy can assess overall bone marrow cellularity and architecture, and help differentiate MDS from myeloproliferative disorders (reticulin deposits and fibrosis). A bone marrow biopsy in MDS usually shows hypercellular marrow. Hypocellular marrow may be seen in some patients, but this is rare. The diagnosis of MDS is based on morphological features, supported by flow cytometric changes and molecular mutations. In the absence of genetic mutations, the morphological features alone should be interpreted with care. Management is stratified by comprehensive risk assessment, specific disease subtype, the severity of cytopenias, and endogenous erythropoietin levels. Supportive care plays an important role in treatment for all patients, and includes transfusions of red blood cells with iron chelation, platelet transfusions, and antibiotics [5].

There are studies regarding the haematological manifestations of hyperparathyroidism. The recognition of the potential association between anaemia and PHPT dates back to the 1930s [6]. Research conducted in India linked PHPT to anaemia and bone marrow fibrosis. This study analysed 28 patients with symptomatic PHPT and found that 15 patients (53%) had anaemia. Bone marrow examination in eight of them revealed bone marrow fibrosis in six patients (21%). It was observed that both anaemia and marrow fibrosis improved after successful parathyroidectomy, and this corrective response was sustained in the same cohort of eight patients at the 10-year follow-up [7,8]. Thrombocytopenia was reported by Bhadada et al. in 2018 [9] and by De Keukeleire et al. in 2017 [10]. Importantly, both cases observed the reversal of thrombocytopenia following parathyroidectomy. 

A larger study evaluated the haematological manifestations of PHPT in a sample of 134 patients with PHPT who underwent surgery for parathyroid adenomas. The study reported leucopenia in 9 out of 134 patients (6.7%), and the resolution of leucopenia was noted in six of these nine patients after parathyroidectomy [11]. However, the study was a retrospective review of blood results after parathyroidectomy, without evidence of bone marrow biopsy data, thus limiting the pathological explanation of the findings. 

Existing case reports of pancytopenia in hyperparathyroidism describe features of bone marrow fibrosis on biopsy, with a similar recovery of cell counts following parathyroidectomy. This resolution has been noted in teenagers, as reported by Kumbasar et al. in 2004 [12] and Akyay et al. in 2013 [13]. A similar response in adults in their 40s has also been reported by Lim et al. [14], Opie et al. [15], and Rajan et al. [16]. 

In our case, neutropenia was the only affected cell line, without anaemia or thrombocytopenia. The bone marrow biopsy showed morphological evidence of myelodysplasia, without features of fibrosis. The remarkable recovery of neutrophils after parathyroidectomy mirrors other existing case reports, which show complete recovery of pancytopenia. However, it is important to note that a follow-up bone marrow biopsy was not performed post-parathyroidectomy; therefore, we cannot confirm whether the morphological changes have resolved or not. As such, the direct causality of the neutropenia cannot be fully established despite the strong temporal association, especially given the unique presentation of isolated neutropenia with concomitant myelodysplasia in the absence of bone marrow fibrosis, for which no similar case has been reported. 

We also considered the following differential diagnoses. Autoimmune-induced neutropenia was a possibility, given the medical history of type 1 diabetes. Drug-induced neutropenia was also a reasonable differential diagnosis, considering the use of acyclovir and co-trimoxazole for prophylaxis, as well as the use of obinutuzumab as maintenance therapy for follicular lymphoma. However, the complete and sustained resolution of the neutropenia after surgical correction of PHPT suggests this is the most likely explanation, as such a response would not be expected in cases of autoimmune or drug-induced neutropenia after the causative agents were discontinued. 

## Conclusions

In conclusion, patients with persistent neutropenia may have undiagnosed PHPT, as this is a rare but reversible cause of persistent neutropenia. Although the exact pathophysiological mechanism remains unclear, the remarkable, complete resolution of the neutropenia after surgical correction of PHPT in our patient reaffirms the association. This case highlights that isolated neutropenia can also be associated with PHPT, providing further evidence in addition to existing research and similar case reports that have focused more on anaemia and pancytopenia. Therefore, we recommend investigating refractory neutropenia or pancytopenia with bone profiles, including calcium, phosphate, and PTH levels. Early identification of hyperparathyroidism and prompt referral to the Endocrinology Team can be critical, as surgical intervention can lead to a complete recovery of neutropenia. 
